# Supplementary figures and images for: Signatures of Arithmetic Simplicity in Metabolic Network Architecture
Source: PLoS Comput Biol. 2010 Apr 1;6(4):e1000725. doi: 10.1371/journal.pcbi.1000725 (PMC2848538; doi:10.1371/journal.pcbi.1000725)

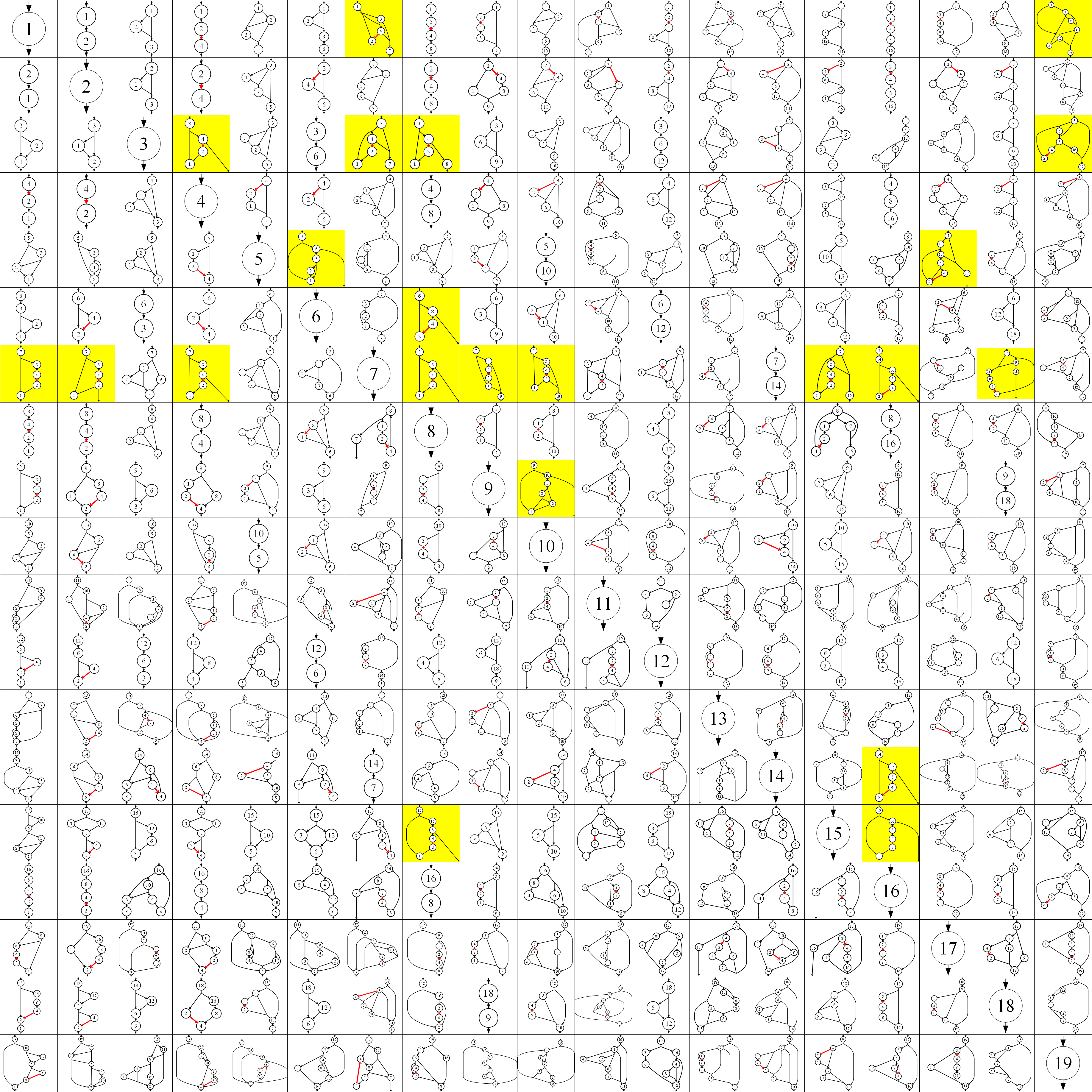

Supplement: Figure S1 — A version of Fig. 2, extended to the R19 network. As in Fig. 2, yellow boxes denote autocatalytic cycles; the red reaction is the one that is used most often, a2 + a2 < = > a4. (2.81 MB TIF) [file pcbi.1000725.s001.tif]
