# Supplementary material for: Signatures of Arithmetic Simplicity in Metabolic Network Architecture
Source: PLoS Comput Biol. 2010 Apr 1;6(4):e1000725. doi: 10.1371/journal.pcbi.1000725 (PMC2848538; doi:10.1371/journal.pcbi.1000725)
Supplement: Figure S2 — Heatmaps describing MBP length and degeneracy (0.02 MB PDF) [file pcbi.1000725.s002.pdf]

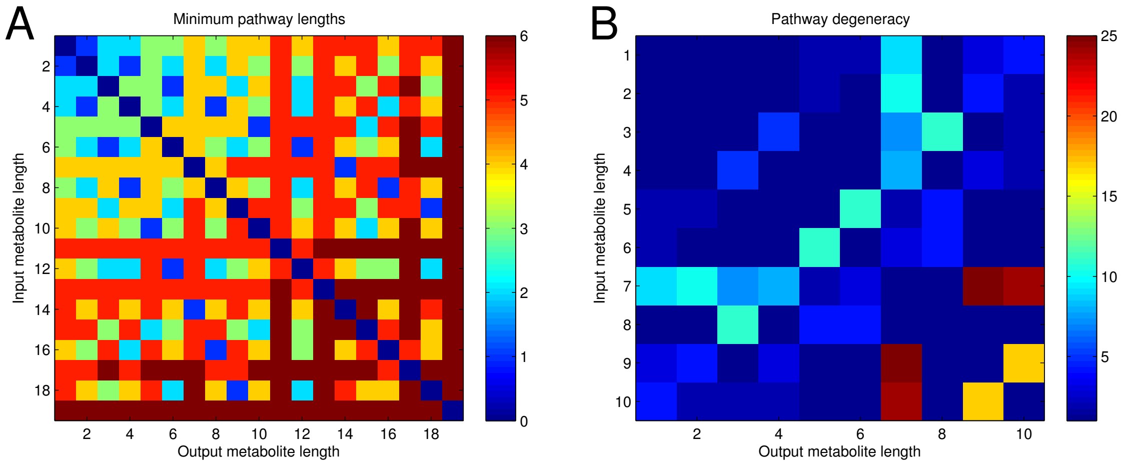

Figure S2 (**A**) This heatmap matrix shows the number of reactions used in all MBPs in the R_{19} network. Each element (i, j) shows the length of the shortest path from a_i to a_j, not counting the transport reactions to allow entry of a_i or exit of a_j. For example, element (1, 1) ($a_1 \Rightarrow a_1$) requires 0 reactions, while (1, 19) ($a_1 \Rightarrow a_{19}$) requires 6 for a minimal pathway. (**B**) This heatmap shows how many MBPs with an equal number of reactions were calculated in the R_{10} network using the EFM method. Each element (i, j) shows how many MBPs exist to convert a_i to a_j. For example, There is only one path to convert a_1 to a_2, but 25 that convert from a_7 to a_9.
