# Supplementary material for: Signatures of Arithmetic Simplicity in Metabolic Network Architecture
Source: PLoS Comput Biol. 2010 Apr 1;6(4):e1000725. doi: 10.1371/journal.pcbi.1000725 (PMC2848538; doi:10.1371/journal.pcbi.1000725)
Supplement: Figure S3 — Logarithmic trend of metabolic pathway length (0.04 MB PDF) [file pcbi.1000725.s003.pdf]

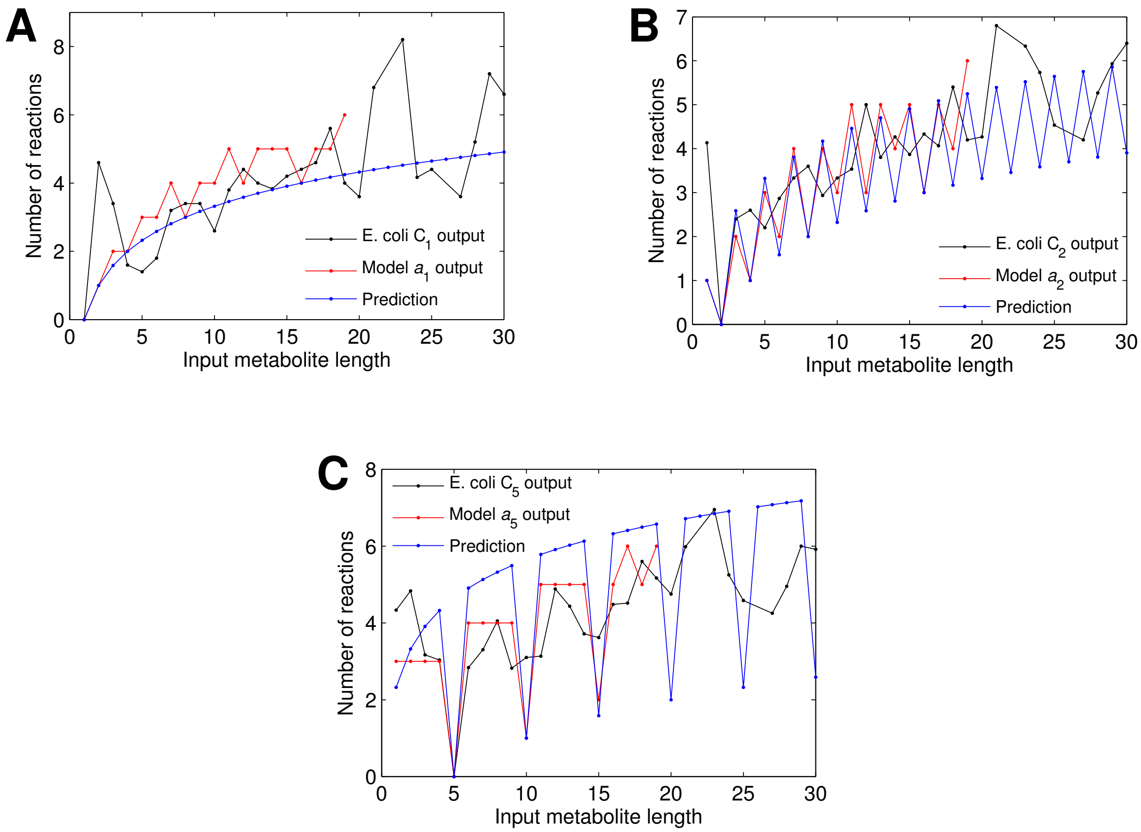

Figure S3 As in Figure 3, we see logarithmic growth among pathways from the R_{19} network and the *E. coli* metabolic network. In this figure, each curve represents the pathway length or number of reactions (y-axis) from progressively growing starting compounds (x-axis) to a given target compound. The red lines show the number of reactions in each MBP with a different input metabolite size, and the blue lines show the number of reactions predicted by equation (1) from the text. The black lines show the average number of reactions to reach the target from the nearest starting compound – similar to method used in Figure 3. (**A**) C_1/a_1 output. (**B**) C_2/a_2 output. (**C**) C_5/a_5 output.
