# Supplementary material for: Signatures of Arithmetic Simplicity in Metabolic Network Architecture
Source: PLoS Comput Biol. 2010 Apr 1;6(4):e1000725. doi: 10.1371/journal.pcbi.1000725 (PMC2848538; doi:10.1371/journal.pcbi.1000725)
Supplement: Figure S4 — Metabolite and reaction usage frequencies for KEGG hydrogen, nitrogen, and oxygen sets (0.09 MB PDF) [file pcbi.1000725.s004.pdf]

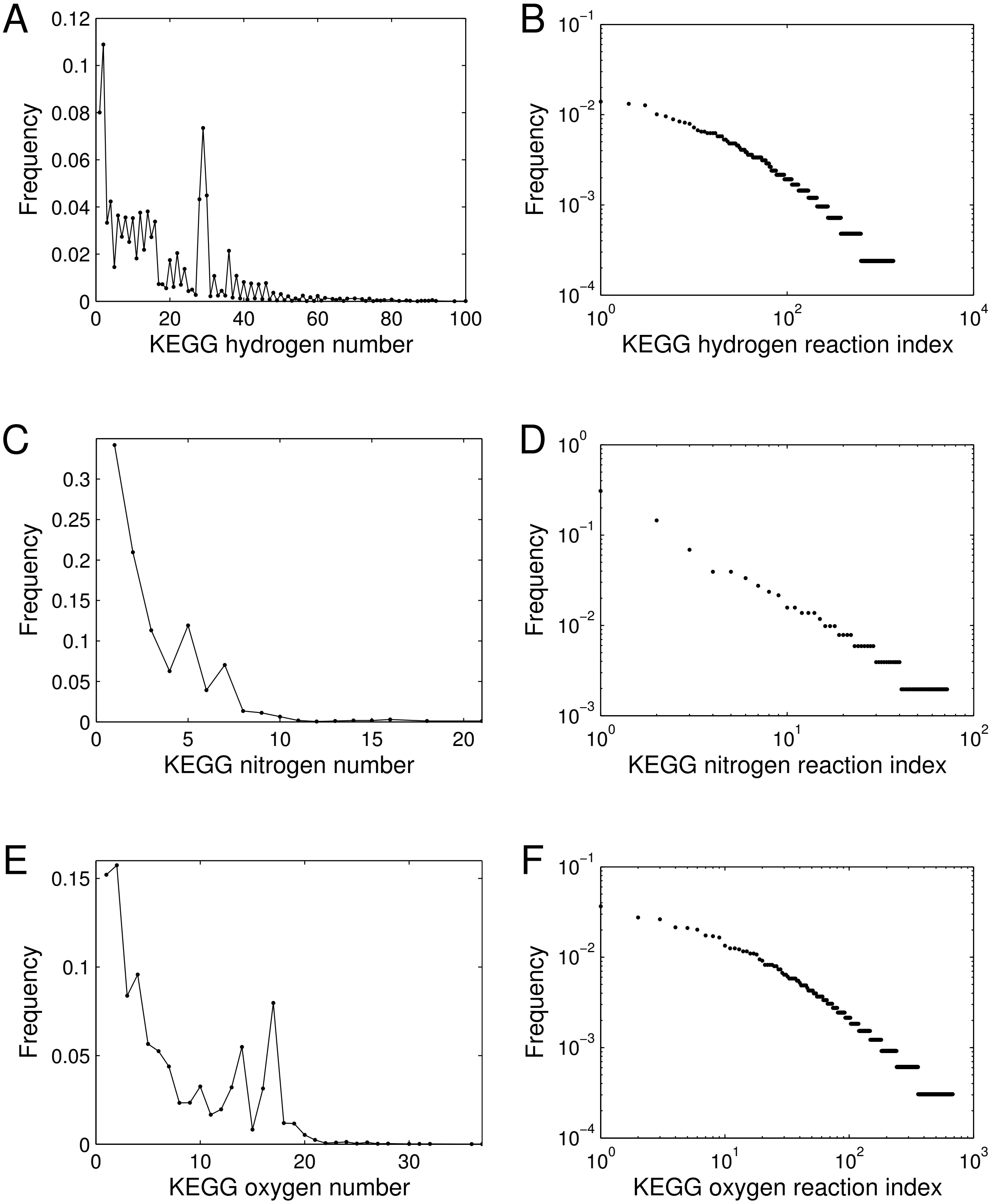

Figure S4 Distributions of reaction and metabolite usage from the KEGG hydrogen, nitrogen, and oxygen –only networks. Calculation of each of these distributions was done by counting the number of times each metabolite (or reaction) appeared in the respective reduced KEGG network. In each of the reaction plots (**B**, **D**, **F**), the values are sorted by rank and shown on log-scale axes. (**A**) Distribution of metabolite usage from the

hydrogen network. (**B**) Ranked distribution of reaction usage from the hydrogen network. The tail follows a power law with exponent -0.79 (R^2 = 0.98). (**C**) Metabolite usage from the nitrogen network. (**D**) Reaction usage from the nitrogen network. The tail follows a power law with exponent -1.20 (R^2 = 0.99) (**E**) Metabolite usage from the oxygen network. (**F**) Reaction usage from the oxygen network. The tail follows a power law distribution with exponent -1.01 (R^2 = 0.99).
