# Supplementary material for: Signatures of Arithmetic Simplicity in Metabolic Network Architecture
Source: PLoS Comput Biol. 2010 Apr 1;6(4):e1000725. doi: 10.1371/journal.pcbi.1000725 (PMC2848538; doi:10.1371/journal.pcbi.1000725)
Supplement: Figure S5 — Fast Fourier transforms showing periodicity of metabolite usage (0.02 MB PDF) [file pcbi.1000725.s005.pdf]

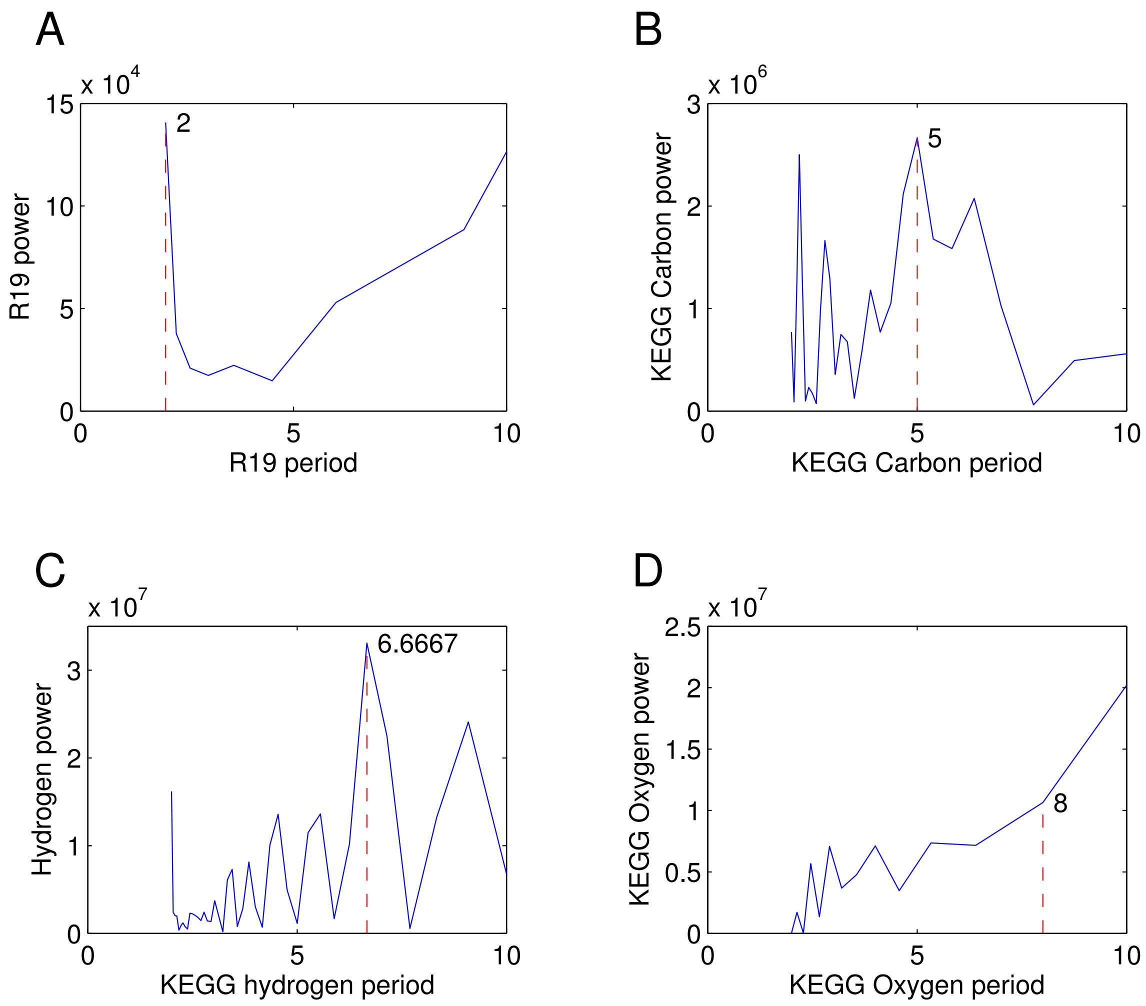


**Figure S5** A series of fast Fourier transforms to detect the period of metabolite usage in the $R_{19}$, KEGG carbon, nitrogen, and oxygen distributions. The highest peak of each period is labeled with the value. (**A**) $R_{19}$ model. (**B**) KEGG carbon. (**C**) KEGG hydrogen. (**D**) KEGG oxygen.
