# Supplementary material for: Signatures of Arithmetic Simplicity in Metabolic Network Architecture
Source: PLoS Comput Biol. 2010 Apr 1;6(4):e1000725. doi: 10.1371/journal.pcbi.1000725 (PMC2848538; doi:10.1371/journal.pcbi.1000725)
Supplement: Figure S6 — KEGG carbon reaction and metabolite usage with cofactors removed PDF (0.04 MB PDF) [file pcbi.1000725.s006.pdf]

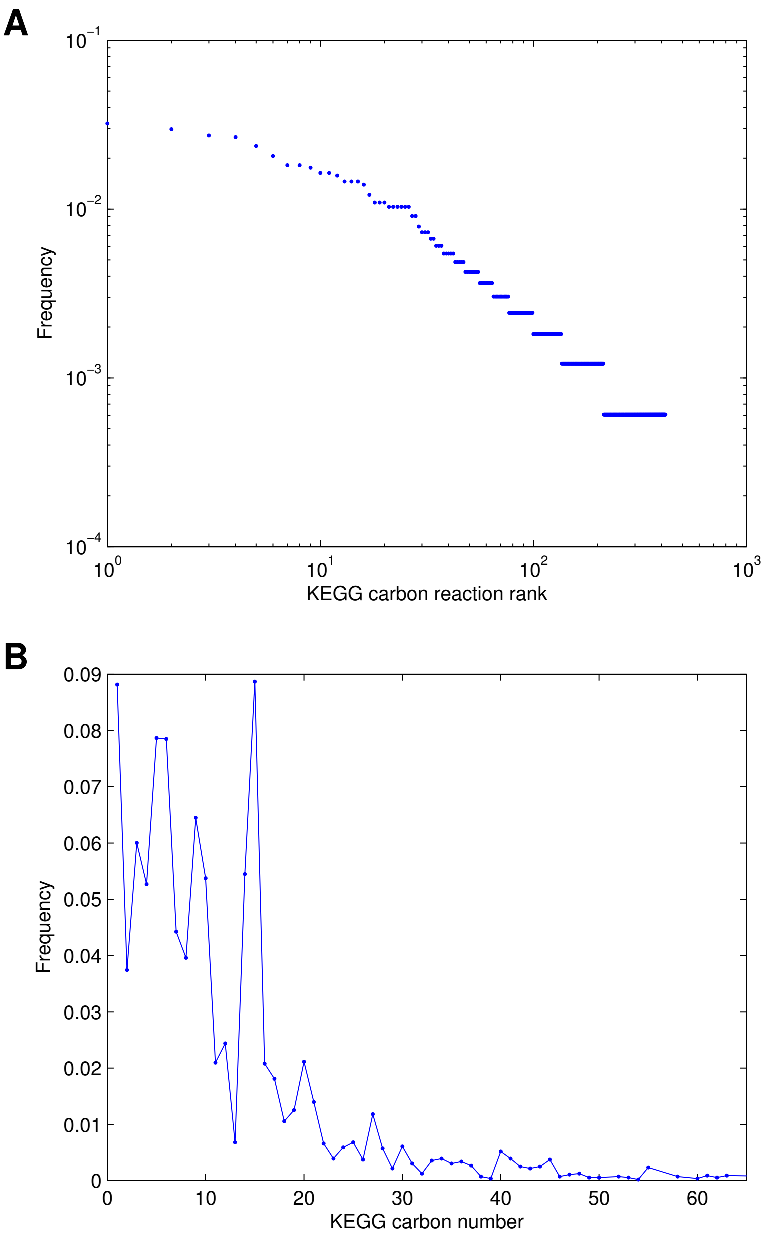

Figure S6 The reaction and metabolite usage of the KEGG-derived carbon network after removal of certain cofactors (listed in Methods). (**A**) The ranked usage of each reaction. As in figures 4 and S4, the usage of each reaction was ranked from greatest to least and sorted accordingly. This gives a long-tailed distribution in which a few reactions are used very often, but many are seldom used. (**B**) The usage of each metabolite. As in Figure 4, the usage of each carbon-number metabolite is calculated by the number of times it shows up among all reactions. Removal of the cofactors causes the periodicity of use of each carbon number to shift slightly to ~4.5 (by fast-Fourier transform, data not shown).
