# Supplementary material for: Signatures of Arithmetic Simplicity in Metabolic Network Architecture
Source: PLoS Comput Biol. 2010 Apr 1;6(4):e1000725. doi: 10.1371/journal.pcbi.1000725 (PMC2848538; doi:10.1371/journal.pcbi.1000725)
Supplement: Figure S7 — Pathway length comparison between iterative algorithm and algorithm minimizing absolute values of fluxes (0.06 MB PDF) [file pcbi.1000725.s007.pdf]

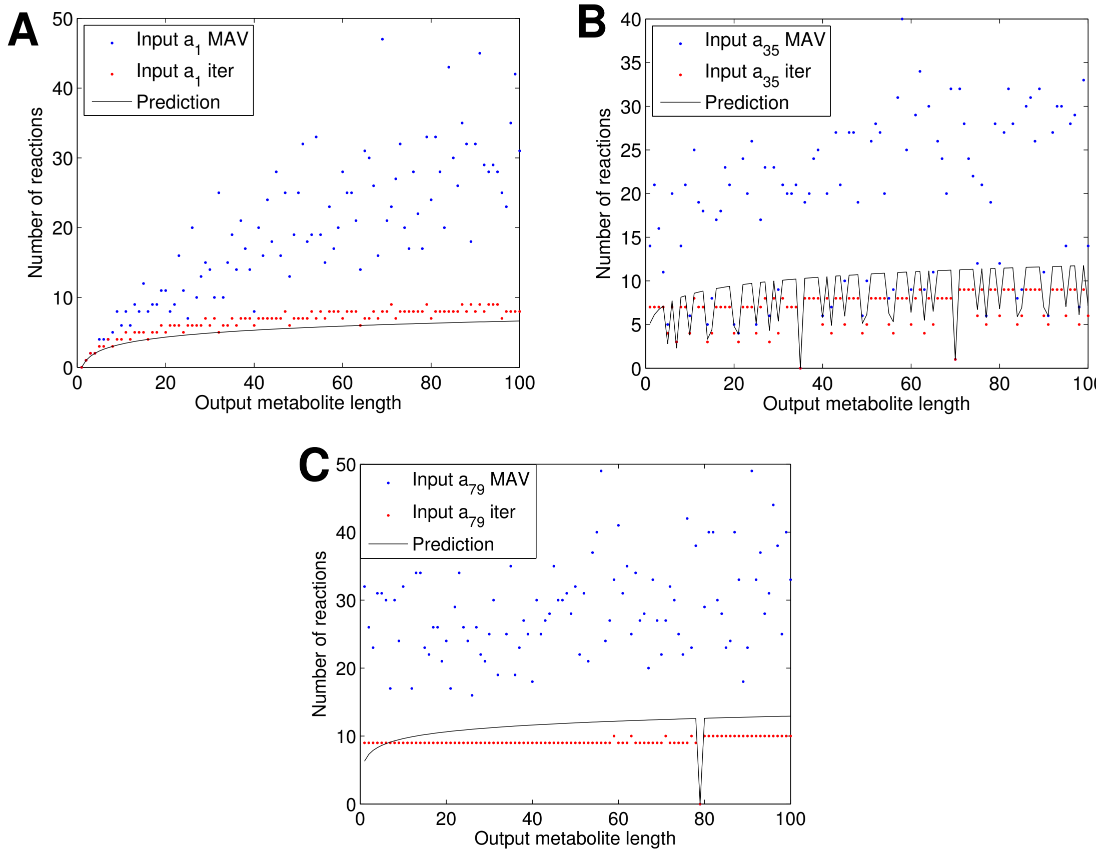

Figure S7

We compared two methods that may approximate the flux balance pathways with the fewest number of active reactions. The first of these uses a standard FBA method in which we minimize the absolute values of all fluxes (MAV, blue points). The second is an algorithm that iteratively produces pathways based on longer input and output metabolites based on solutions to problems involving shorter metabolites (iter, red points, see Methods). These are compared to the predicted minimal solutions given by equation (1) of the text (Prediction, black line). Both of these can be efficiently produced for all metabolites in very large networks – the R_{100} network was used for this analysis. We show in this figure representative results of the resulting lengths of pathways in which a single input was used to construct all 100 outputs. It is clear from this analysis that of the two heuristics, the iterative algorithm best approximates the optimal metabolic pathways. (**A**) Input a_1, (**B**) Input a_{35}, (**C**) Input a_{79}.
