# Supplementary material for: Signatures of Arithmetic Simplicity in Metabolic Network Architecture
Source: PLoS Comput Biol. 2010 Apr 1;6(4):e1000725. doi: 10.1371/journal.pcbi.1000725 (PMC2848538; doi:10.1371/journal.pcbi.1000725)
Supplement: Figure S8 — Reaction usage is similar between R10 EFM and MILP results (0.02 MB PDF) [file pcbi.1000725.s008.pdf]

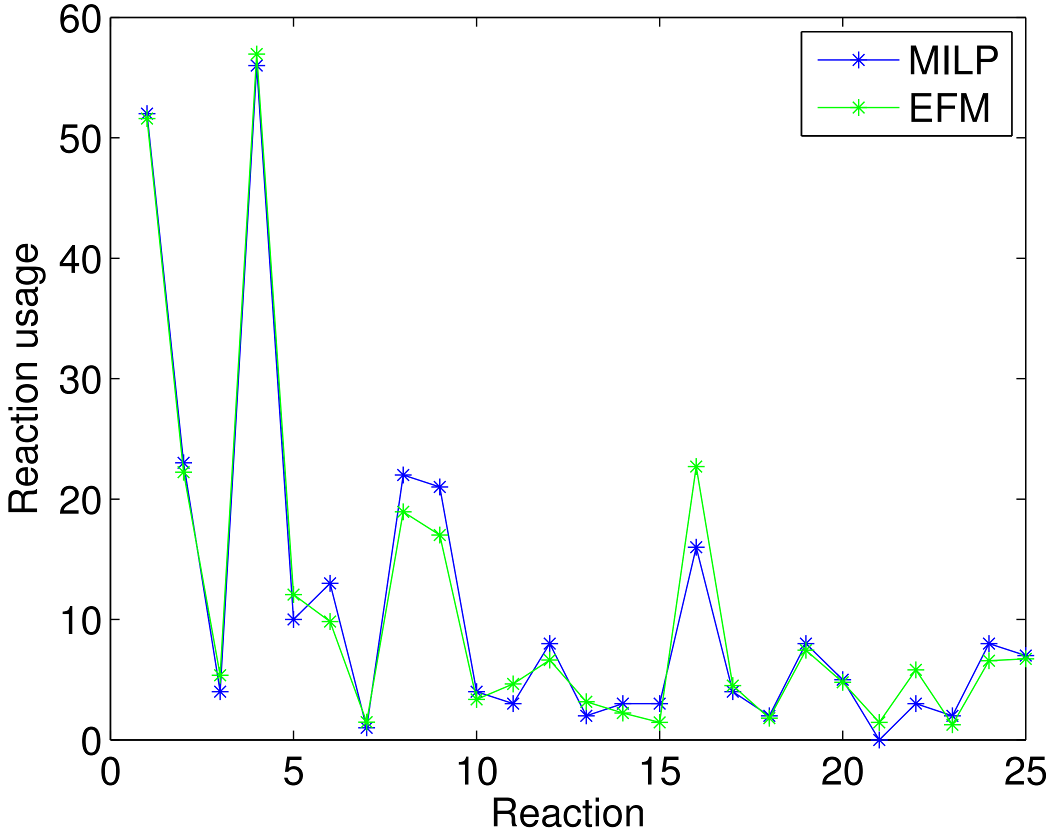

Figure S8 We analyzed the difference in MBP reaction usage between a set of single metabolic network solutions yielded by two algorithms in the R_{10} network. The MILP algorithm (MILP, blue line) and the set of solutions gathered from the elementary flux modes (EFM, green line) are compared here. For the set of EFMs, each case of a single input leading to a single output often resulted in several equally optimal networks. The reaction use of each of these degenerate sets was averaged, to ensure that the pathways of each $a_i \Rightarrow a_j$ problem contributed equally to the reaction usage distribution. The x-axis shows the index of each individual reaction in the R_{10} network (in arbitrary order), and the y-axis shows how often each reaction is used across MBPs. These distributions are not significantly different (p-value = 0.031, Wilcoxon rank-sum test), thus the reaction distribution from the MILP algorithm is as effective as that of the EFM method.
