# Supplementary material for: Signatures of Arithmetic Simplicity in Metabolic Network Architecture
Source: PLoS Comput Biol. 2010 Apr 1;6(4):e1000725. doi: 10.1371/journal.pcbi.1000725 (PMC2848538; doi:10.1371/journal.pcbi.1000725)
Supplement: Figure S9 — Removing reactions from the R19 network affects the system's ability to yield balanced pathways (0.04 MB PDF) [file pcbi.1000725.s009.pdf]

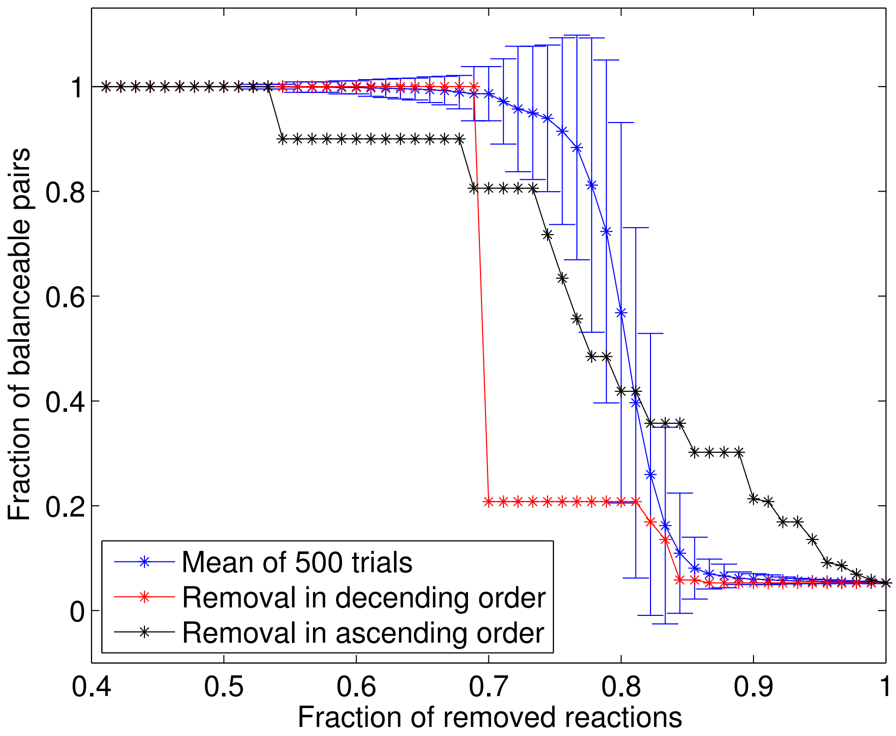

Figure S9 The importance of the most highly used reactions in terms of their capacity to produce given outputs was analyzed by progressively removing reactions from the R_{19} network, then calculating how the number of FBA solutions changed. We determine this number by calculating how many $a_i \Rightarrow a_j$ trials could yield a valid, balanced set of fluxes using FBA. This was performed in three different cases. The first (blue line) involved randomly removing reactions. Reactions were removed one at a time, then each $a_i \Rightarrow a_j$ (for $1 \leq i, j \leq 19$) was solved using a standard FBA routine. This was repeated 500 times, and the average curve is shown (error bars represent a single standard deviation). The fraction of solvable pairs remains high until approximately 70% of reactions are removed from the network, at which point the fraction falls off sharply. This is in contrast to the trial in which the reactions are removed in descending order (e.g., the most highly used reactions are removed first, red line). Although all $a_i \Rightarrow a_j$ problems again remained solvable until 70% of reactions were removed, the fraction dropped off far more sharply than the average. Conversely, the fraction of solvable problems was much more gradual when reactions were removed in ascending order (the least used reactions were removed first, black line). This implies that, regardless of the output produced, there are reactions that are more generally applicable than others.
