# Supplementary material for: Signatures of Arithmetic Simplicity in Metabolic Network Architecture
Source: PLoS Comput Biol. 2010 Apr 1;6(4):e1000725. doi: 10.1371/journal.pcbi.1000725 (PMC2848538; doi:10.1371/journal.pcbi.1000725)
Supplement: Table S1 — Comparison of the three different MBP search algorithms (0.04 MB DOC) [file pcbi.1000725.s010.doc]

**Table S1**

Comparison of the three different MBP search algorithms.

|  | **MILP** | **EFM** | **Iterative** |
| --- | --- | --- | --- |
| **Strengths** | Provides an exact FBA solution, using tools previously described for FBA. | Provides all degenerate solutions for a given input/output pair. | Can provide multiple degenerate solutions for network size of up to R100 or larger |
| **Weaknesses** | Limited to the R19 network, and only provides a single optimal solution. | Limited to the R10 network due to memory requirements and the rapidly growing complexity inherent to any RN network. | Cannot calculate autocatalytic cycles, and because it bases each progressive pathway on the previous optimal pathway, it can miss some pathways that should be based on sub-optimal precursors. |
